# Meta-analysis of the association between dietary inflammatory index and cognitive health

**DOI:** 10.3389/fnut.2023.1104255

**Published:** 2023-04-04

**Authors:** Tianze Ding, Maimaitiyusupu Aimaiti, Shishuang Cui, Junhao Shen, Mengjie Lu, Lei Wang, Dongsheng Bian

**Affiliations:** ^1^Department of Geriatrics, Medical Center on Aging of Shanghai Ruijin Hospital, Shanghai Jiao Tong University School of Medicine, Shanghai, China; ^2^Department of Nutrition, College of Health Science and Technology, Shanghai Jiao Tong University School of Medicine, Shanghai, China; ^3^Department of Geriatrics, Ruijin Hospital, Shanghai Jiao Tong University School of Medicine, Shanghai, China; ^4^School of Public Health, Shanghai Jiao Tong University School of Medicine, Shanghai, China; ^5^Department of Clinical Nutrition, Ruijin Hospital, Shanghai Jiao Tong University School of Medicine, Shanghai, China

**Keywords:** cognitive health, mild cognitive impairment, dietary inflammatory index, cognitive impairment, pro-inflammatory diet

## Abstract

**Background:**

Some studies have shown that a pro-inflammatory diet may be associated with cognitive function, but their conclusions have varied considerably. We here present a meta-analysis of the current published literature on DII score and its association with cognitive health.

**Methods:**

In this meta-analysis, the PubMed, Embase, Web of Science, and Cochrane databases were searched in September 2022. The reported indexes, specifically OR, RR, and β, were extracted and analyzed using R version 3.1.0.

**Results:**

A total of 636 studies in databases were identified, and 12 were included in the meta-analysis. Higher DII was associated with an increased risk of AD and MCI (OR = 1.34; 95% CI = 1.21–1.49). Meanwhile, it may also cause global function impairment (categorical: OR = 1.63; 95% CI = 1.36–1.96) and verbal fluency impairment (continuous: OR = 0.18; 95% IC = 0.08–0.42). But there was no significant association between DII and executive function (categorical: OR = 1.12; 95% IC = 0.84–1.49; continuous: OR = 0.48; 95% IC = 0.19–1.21) or episodic memory (continuous: OR = 0.56; 95% IC = 0.30–1.03).

**Conclusion:**

A pro-inflammatory diet is related to AD, MCI, and the functions of some cognitive domains (specifically global function and verbal fluency). However, the current evidence on the role of diet-induced inflammation in different cognitive domains should be supported by further studies in the future.

## Introduction

The aging of today’s population has become a critical challenge worldwide. The proportion of the elderly population has been gradually increasing. By 2030, the proportion of people aged 60 and over may increase from 1 billion to 1.4 billion. By 2050, the world’s population aged 60 and older may double (2.1 billion) (1). Many issues come with this demographic shift, including increased rates of cognitive decline and dementia. According to WHO, there are 55 million people with dementia at present around the world, and nearly 10 million new cases annually (2). The most common form of dementia is Alzheimer’s disease, which may cause 60–80% of cases (3). Alzheimer’s disease and other types of dementia are reported to be the seventh leading cause of death among the elderly and some of the most common causes of disability worldwide (2). Mild cognitive impairment (MCI) is considered a precursor stage of dementia. One study found that 32% of patients with mild cognitive impairment progressed to dementia within 5 years (4), so that effective measures to prevent such cognitive impairment are essential.

Inflammation has been confirmed to be one possible mechanism underlying cognitive impairment (AD, dementia, and MCI) in recent studies (5, 6). Whether it is dietary patterns, dietary composition, or compounds in the diet, diet seems to be related to the level of inflammation in the body (7, 8). From this perspective, diet might affect cognition by modulating inflammation in the body. Some studies have investigated the association between diet and cognitive health, and their findings suggest that different diets increase (8) or decrease (9) the risk of cognitive impairment. Establishing a means of quantitatively measuring the inflammatory potential of diet would allow observation of a direct and dynamic link between the ability of diet to modulate levels of inflammation *in vivo* and cognitive health. This would make it possible to prevent or delay cognitive impairment with specific modifications to dietary factors within a flexible range.

In order to determine the inflammatory potential of a given diet, Shivappa et al. proposed a dietary inflammatory index (DII), which includes 36 anti-inflammatory parameters for fiber, alcohol, monounsaturated fatty acids, polyunsaturated fatty acids, omega 3, omega 6, niacin, thiamin, riboflavin, vitamin B6, B12, zinc, magnesium, selenium, vitamin A, vitamin C, vitamin D, vitamin E, folic acid, β carotene, anthocyanidins, flavan-3-ols, flavonols, flavanones, flavones, isoflavones, garlic, ginger, onions, thyme, oregano, saffron, turmeric, rosemary, eugenol, caffeine and tea, and 9 pro-inflammatory parameters for energy, carbohydrates, proteins, total fat, trans fat, cholesterol, vitamin B12, saturated fatty acids, and iron, for a total of 45 parameters. Individuals’ diets were classified along the DII according to inflammatory potential on a continuum from maximum pro-inflammatory to maximum anti-inflammatory. The higher the DII score, the greater the inflammatory potential of the given diet (10).

Existing epidemiological studies have explored the association between DII and cognitive impairment (MCI, dementia, and AD) and cognitive function (global cognition, episodic memory, executive function, verbal fluency, tested using mini-mental state examination (MMSE), or other common cognitive function tests such as the digit span-backward test or MoCA-score). All studies on the relationship between DII and the incidence of MCI or AD showed a positive relationship (11–15). Some studies showed a negative relationship between DII and global cognition and verbal fluency (12, 14, 16–20). Other studies searched for a relationship between DII and episodic memory and executive function and found none (19–21). Some systematic reviews have focused on the effects of DII on mental health (22, 23) and the influences of dietary factors on cognitive health (9, 24), but, as far as we know, no systematic review has investigated the effects of DII on cognitive disorders or cognitive function. Given this, we performed a meta-analysis to comprehensively examine the association between DII and cognitive health including cognitive disorders (impairment) and cognitive function.

## Methods

This meta-analysis was performed in accordance with the PRISMA statement to ensure comprehensive and transparent reporting of methods and results.

### Search strategy

The literature databases PubMed, EMBASE, Web of Science, and Cochrane were searched in September 2022 and the search terms used were “mild cognitive impairment” OR dementia OR “Alzheimer disease” OR “cognitive function” OR “cognitive decline” AND “dietary inflammatory index” OR “anti-inflammatory diet” OR “inflammatory diet.” Two authors independently screened each title and abstract and then read the full text to assess the article for compliance with the inclusion and exclusion criteria which will be mentioned later. Disagreements were resolved through consensus.

### Selection criteria

Studies included in this meta-analysis met all the following inclusion criteria: (1) studies that focused on humans and were published in English; (2) investigated the association between the DII/E-DII and cognitive function (global cognition, episodic memory, executive function, verbal fluency) or the risk of cognitive disorders (MCI, dementia, AD); (3) the study design was case–control, cohort or cross-sectional study. (4) If multiple studies were published using the same cohort, only the most recent study was selected. If more than one article based on the same cohort was from a different population or reported different results, both were included. Studies were excluded if they were systematic reviews or narrative reviews, reports, letters, *in vitro* studies, animal studies or duplicate studies.

### Data extraction

Two authors (TD and MA) screened the papers and abstracted the data independently, and conflicts were resolved by a third reviewer (DB) who was blinded to the authors and institutions of the studies undergoing review. The following information was extracted from each study: name of the first author, year of publication, country, study design, sample size, study period, dietary assessment method, number of food parameters, cognitive assessment methods, cognitive domains, results, and covariates used for adjustment.

### Statistical analysis

In this meta-analysis, all the reported effects, odds ratios (ORs), hazard ratios (HRs), risk ratios (RRs) and β with 95% confidence intervals and effect size were extracted. The heterogeneity test was assessed using Cochrane’s *Q* test and the *I*^2^statistic. Significance was defined as *p* < 0.05. The *I*^2^ statistic represents the amount of total variation that could be attributed to heterogeneity. *I*^2^ values ≤25%, ≤50%, ≤75%, and >75% indicated no, little, moderate, and high heterogeneity, respectively. For lower heterogeneity (*I*^2^ ≤ 50%), the fixed effect models were used, and random effect models were used for higher heterogeneity (*I*^2^ > 50%). A sensitivity analysis had been conducted by excluding one study at a time to identify potential sources of heterogeneity when the result had a high heterogeneity (*I*^2^ > 50%). The meta-analysis was performed using the “*meta*” package in R. Statistical analyses were performed using R version 3.1.0 (R Foundation for Statistical Computing, Vienna, Austria).

## Results

### Literature selection

**Figure 1:**
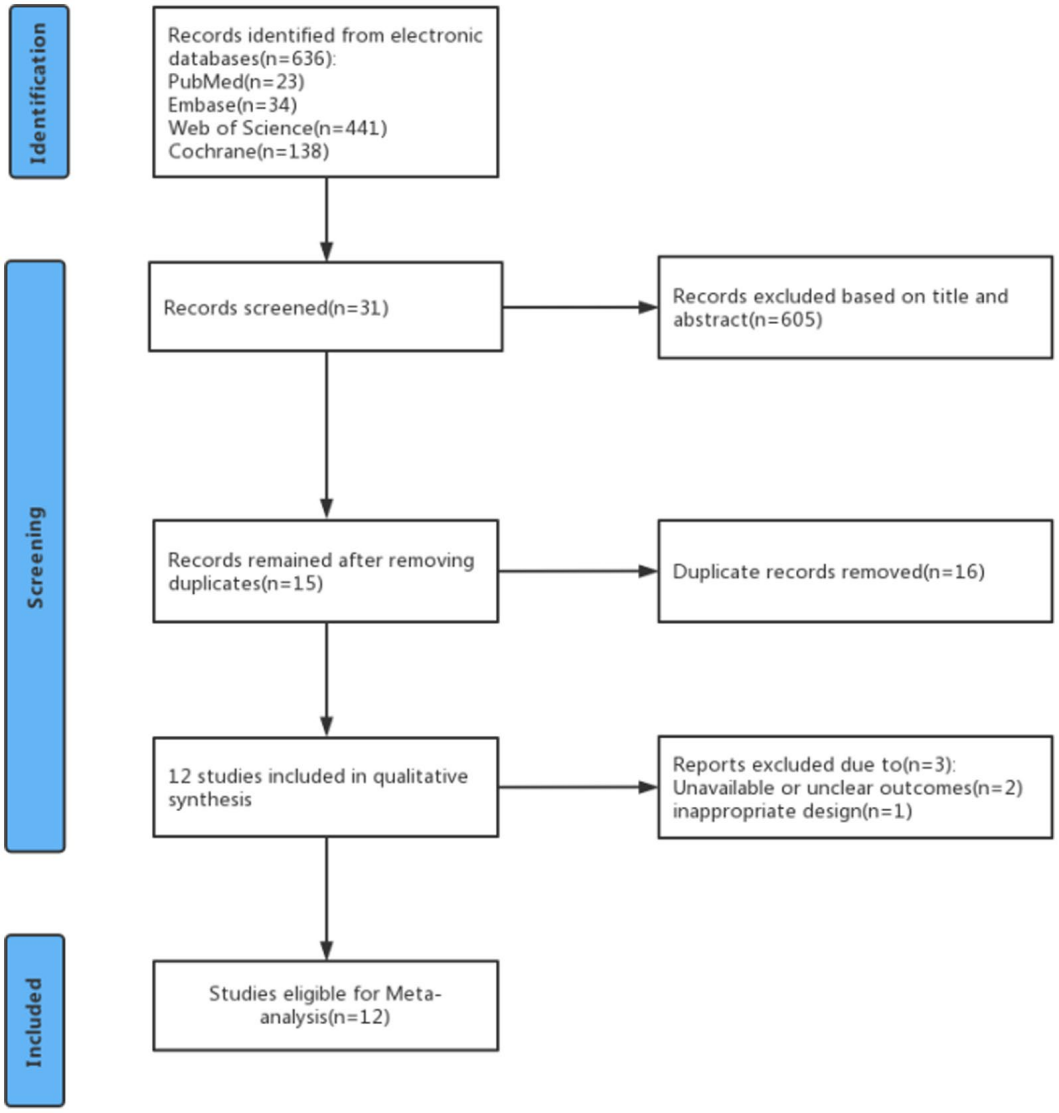
Study identification and selection.

A total of 636 studies were identified in the PubMed, Embase, Web of Science, and Cochrane databases. Of these, 605 studies were excluded after reviewing their titles and abstracts to assess relevance and eligibility. Then 16 articles were excluded because they were duplicates. After careful perusal of the full texts of these articles, 3 were excluded because of unavailable or unclear outcomes and inappropriate design. Finally, 12 studies were included in the meta-analysis (Figure 1).

### Study characteristics

The characteristics of the included studies are summarized in Table 1. Of these studies (11–21, 25) were published between 2017 and 2022, and their sample sizes varied from 222 to 7,085 participants with a total number of 24,823 individuals from seven countries on four continents. Four studies were carried out in the United States, three studies (12–14) in China, and one each in France (20), Greece (15), Poland (17), Australia (25), and South Korea (16). The average age of participants varied from 61.00 to 74.04. In terms of dietary assessment, six studies (11–15, 25) used Food Frequency Questionnaire (FFQ), five studies (16, 18–21) used 24-h recall, and only one study (17) used three-day food diaries. Regarding the number of food parameters used to calculate DII score, two studies (15, 17) used all 45 parameters; three studies (18, 21, 25) used the E-DII score, which contains 27 parameters; and other studies varied between 22 and 35 parameters. More detailed summaries of each article are included in Table 2, which provides information regarding assessment instruments for cognitive function and results for each study. Cognitive function was assessed using a large number of tests to quantify the cognitive domains of global cognition, episodic memory, executive function, and verbal fluency. There were differences among individual studies. For example, studies used different assessment instruments, including MoCA, MMSE, Petersen’s Criteria, CERAD, DSM-IV-TR criteria, DSST, Animal Fluency Test, and RI-48.

**Table 1:** Main characteristics of the included studies.

	Author (Ref.)	Study location	Study design	Study period	Study instrument	Number of food parameters	Sample size	Average age
1	Wang et al. (13)	China	Cross-sectional	2020 ~ 2021	33-item FFQ	23	1,050	70.00
2	Zhang et al. (14)	China	Prospective cohort	2014 ~ 2018	FFQ	24	2,239	58.80
3	Hayden et al. (11)	America	Prospective cohort	1996 ~ 2008	122-item FFQ	32	7,085	71.00
4	Frith et al. (19)	America	Cross-sectional	2011 ~ 2014	24-h recall	26	1723	68.40
5	Kesse-Guyot et al. (20)	France	Prospective cohort	1994 ~ 2009	24-h recall	35	3,080	65.40
6	Sun et al. (18)	America	Cross-sectional	2011 ~ 2014	24-h recall	27	1,198	69.30
7	Charisis et al. (15)	Greece	Prospective cohort	2011 ~ 2022	FFQ	45	1,059	73.10
8	Shin et al. (18)	Korean	Cross-sectional	2012	24-h recall	20	239	74.04
9	Song et al. (21)	America	Cross-sectional	2011 ~ 2014	24-h recall	27	2,901	69.61
10	Liu et al. (12)	China	Cross-sectional	2018 ~ 2019	30-item FFQ	22	3,386	67.61
11	Skoczek-Rubińska et al. (17)	Poland	Cross-sectional	2014 ~ 2020	Three-day food diary	45	222	61.00
12	Zabetian-Targhi et al. (25)	Australia	Cross-sectional	2005 ~ 2011	80-item FFQ	27	641	67.70

**Table 2:** Methods and results of the included studies (by cognitive domain).

Author (Ref.)	Country	Method	*N*	Index	95%CI
**MCI**					
Wang et al. (13)	China	MoCA	1,050	OR = 1.23	1.03, 1.47
Hayden et al. (11)	America	MMSE	7,085	HR = 1.27	1.06, 1.25
Liu et al. (12)	China	Petersen’s criteria (modified)	3,386	OR = 1.595	1.189, 2.140
Zhang et al. (14)	China	MMSE and MoCA	2,239	RR = 1.46	1.14, 1.87
**AD**					
Charisis et al. (15)	Greece	DSM-IV-TR criteria	1,059	HR = 3.01	1.24, 7.26
**Global cognitive**					
Skoczek-Rubińska et al. (17)	Poland	MMSE	222	OR = 11.1	2.22, 55.56
Shin et al. (18)	Korean	MMSE	239	OR = 6.32	1.18, 33.78
Liu et al. (12)	China	Petersen’s criteria (modified)	3,386	OR = 1.595	1.189, 2.140
Zhang et al. (14)	China	MMSE & MoCA	2,239	RR = 1.46	1.14, 1.87
Sun et al. (18)	America	CERAD-WL and AF and DSST	1,198	OR = 1.479	1.009, 2.168
Wang et al. (13)	China	MoCA	1,050	β = −0.363	−0.625, −0.101
Kesse-Guyot et al. (20)	France	Composite cognitive score	3,080	β = −1.76	−2.81, −0.72
Zabetian-Targhi et al. (25)	Australia	A battery of neuropsychological tests	641	β = 0.01	−0.04, 0.06
**Episodic memory**					
Song et al. (21)	America	CERAD-WL	2,901	OR = 1.24	0.87, 1.76
Song et al. (21)	America	CERAD-DR	2,901	OR = 0.93	0.57, 1.51
Frith et al. (19)	America	CERAD	1723	β = −0.39	−0.79, 0.00
Kesse-Guyot et al. (20)	France	RI-48	3,080	β = −1.38	−2.50, −0.27
**Executive function**					
Song et al. (21)	America	DSST	2,901	OR = 1.97	1.08, 3.58
Frith et al. (19)	America	DSST	1,723	β = −2.80	−5.58, −0.02
Kesse-Guyot et al. (20)	France	TMT	3,080	β = −0.61	−1.67, 0.45
Kesse-Guyot et al. (20)	France	Forward digit span tasks	3,080	β = 0.07	−1.05, 1.18
Kesse-Guyot et al. (20)	France	Backward digit span tasks	3,080	β = −0.86	−1.96, 0.25
**Verbal fluency**					
Song et al. (21)	America	AF	2,901	OR = 1.76	1.30, 2.37
Frith et al. (19)	America	AF	1723	β = −1.18	−2.17, −0.20
Kesse-Guyot et al. (20)	France	Phonemic fluency task	3,080	β = −1.42	−2.52, −0.33
Kesse-Guyot et al. (20)	France	Semantic fluency task	3,080	β = −2.57	−3.67, −1.48

MoCA, Montreal Cognitive Assessment; MMSE, Mini-Mental State Examination; CERAD, the Consortium to Establish a Registry for Alzheimer’s Disease: Word Learning/Delayed Recall; AF, Animal Fluency Test; DSST, Digit Symbol Substitution Test; RI-48, Rappel Indicé-48; TMT, Delis–Kaplan trail-making test.

### Meta-analysis

**Figure 2:**
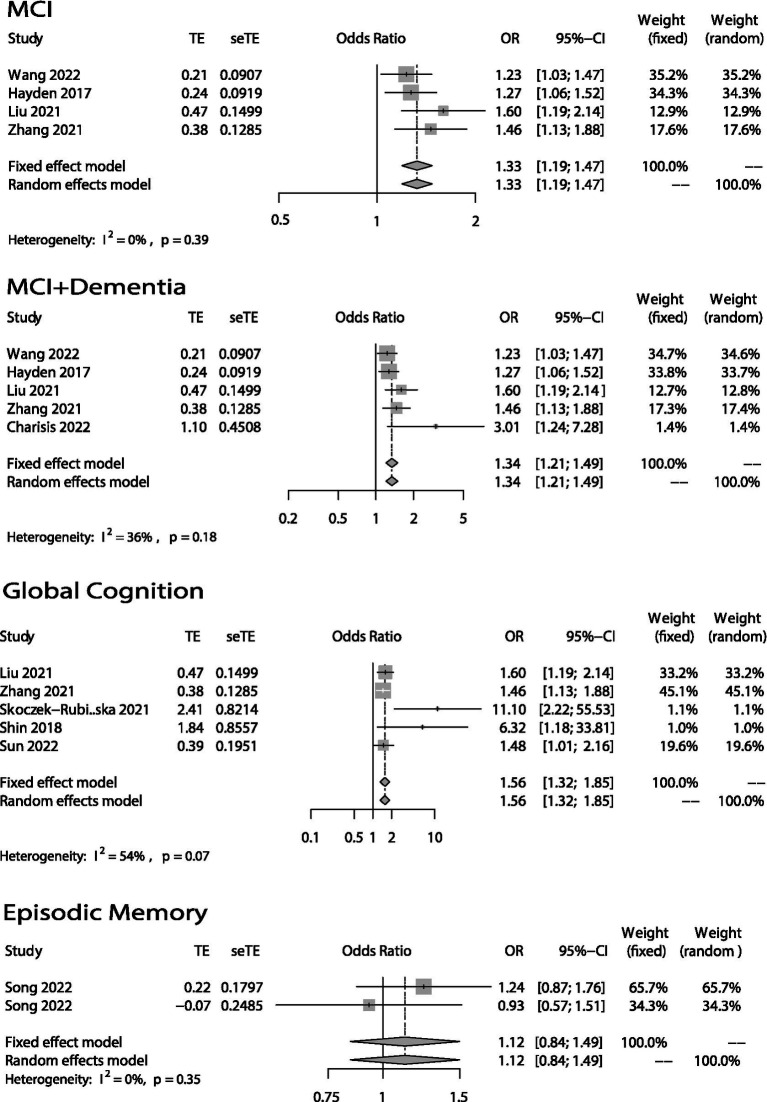
Odds ratios (ORs) of the highest versus lowest category of DII and cognitive health (MCI, MCI + Dementia, Global Cognition and Episodic Memory). Categorical variables.

**Figure 3:**
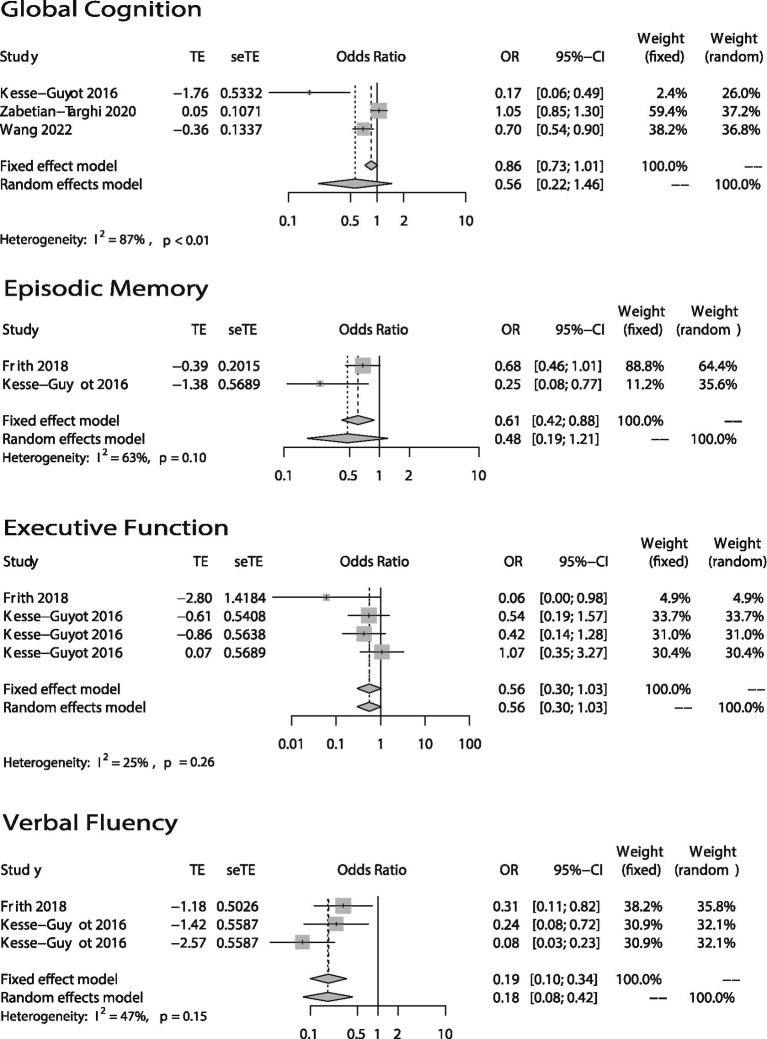
Odds ratios (ORs) of the highest versus lowest category of DII and cognitive health (Global Cognition, Episodic Memory, Executive Function, and Verbal Fluency). Continuous variables.

The forest plots of the meta-analysis were shown in Figure 2 (categorical variables) and Figure 3 (continuous variables). The weight in each plot was calculated by the inverse-variance method (shown below), which meant that if a study had more participants, more events and a narrower confidence interval, it would have a higher weight.

$$\mathrm{Weight}=\frac{1}{\mathrm{variance}\;\mathrm{of}\;\mathrm{estimate}}=\frac{1}{SE^{2}}$$

$$\mathrm{Pooled}\;\mathrm{estimate}=\frac{\mathrm{sum}\;\mathrm{of}\;\left(\mathrm{estimate}\times \mathrm{weight}\right)}{\mathrm{sum}\;\mathrm{of}\;\mathrm{weights}}$$

Four studies (11–14) investigated the association between DII and morbidity rate of MCI (Figure 2). All showed a significant positive association (OR = 1.33; 95% CI = 1.19–1.47) with low heterogeneity (*I*^2^ = 0%, *p* = 0.39). After taking the article (15) which studied the association between DII and the morbidity rate of AD into account, the results did not change much (OR = 1.34; 95% CI = 1.21–1.49).

The association between DII and global cognition was shown in Figures 2, 3. There were eight (12–14, 16–18, 20, 25) studies. And the outcome variables of five of them (12, 14, 16–18) were categorical variables while the other three’s (13, 20, 25) were continuous variables. As to the five studies that used categorical variables, there is a negative association (OR = 1.56; 95% CI = 1.32–1.85) between DII and global cognition with no evidence of statistically significant heterogeneity between the studies (*I*^2^ = 54%, *p* = 0.07). The sensitivity analysis (Supplementary Figure S1) revealed that the result from Skoczek-Rubińska was the largest contributor to the heterogeneity and its exclusion reduced heterogeneity to 0% (*p* = 0.39) with substantially unchanged effect size (OR = 1.53; 95% CI = 1.29–1.81). However, when it comes to the other three studies which used continuous variables, the relationship between DII and global cognition is not significant (OR = 0.56; 95% CI = 0.22–1.46) and heterogeneity is high (*I*^2^ = 87%, *p* < 0.01). The sensitivity analysis (Supplementary Table S1) conducted by excluding one study at a time showed that the heterogeneities between every two articles were high.

Only three studies (19–21) evaluated the relationship between DII and episodic memory (Figures 2, 3). The study (21) that used categorical variables evaluated episodic memory *via* two parts from the same instrument. The results showed that there is no relationship between DII and episodic memory (categorical: OR = 1.12; 95% IC = 0.84–1.49; continuous: OR = 0.48; 95% CI = 0.19–1.21). Both heterogeneities were not high (categorical: *I*^2^ = 0%, *p* = 0.35; continuous: *I*^2^ = 63%, *p* = 0.10).

In terms of the association between DII and executive function, two articles (19, 20) used four methods to evaluate it (Figure 3). Both articles took continuous variables as their outcome variables. Results showed DII has no association with executive function (OR = 0.56; 95% CI = 0.30–1.03) and there was no evidence of statistically significant heterogeneity between the studies (*I*^2^ = 25%, *p* = 0.26).

The association between DII and verbal fluency is shown in Figure 3. The two articles are the articles mentioned above which studied the association between DII and executive function. The results showed that DII score is associated with verbal fluency (OR = 0.18; 95% CI = 0.08–0.42) and the heterogeneity is medium (*I*^2^ = 47%, *p* = 0.15).

## Discussion

This meta-analysis summarizes the evidence underlying associations between DII and cognitive function. High DII was associated with an increased risk of overall cognitive decline, including the morbidity rate of AD and MCI, decline of both global cognition and verbal fluency. However, there was hardly any association detected between DII and either executive function or episodic memory.

The dietary inflammation index (DII) (10) is a quantitative measure of pro-inflammatory diets and is associated with systemic inflammatory markers including tumor necrosis factor (TNF), interleukins (IL), and Interferon gamma (IFN-γ) (10, 26). It was created based on nearly 6,500 studies and has 45 dietary components, including common food items, micronutrients, and important bioactive polyphenols. All these components can affect an individual’s level of inflammation. It is generally acknowledged that neuroinflammation plays an important role in the pathogenesis of Alzheimer’s disease (27, 28) and there are more inflammatory markers (TNFα, IL-1β, IL-6, IL-10) in patients with AD and mild cognitive impairment (MCI) than in healthy controls (29–32). Consistently, previous findings in humans and animals have suggested that neuroinflammation is associated with cognitive decline, especially learning and memory (5, 33). Although we have yet to establish how these inflammatory markers damage our brains, there are some studies that have tried to explain this pathogenesis. One study conducted in Japan (34) suggested that TNFα competes with the cAMP signaling pathway, thereby inhibiting memory retrieval by blocking cAMP-PKA-GluA1 S845 signaling. In this way, TNFα could cause cognitive decline. Moreover, other studies focused on IL-1 found that IL-1β could act directly on hippocampal neurons to interfere with memory reconsolidation and the mice’s working memory impairment was prevented by global IL-1R1 knockout (35–37). Given these links, it is not surprising that we observed such a positive association between pro-inflammatory diets (higher DII) and AD, MCI, and poor global function in the present work.

Among all the forest plots in this article, only the global cognition plot (continuous variables) in Figure 3. Shows high heterogeneity (*I*^2^ = 87%, *p* < 0.01). And the source of heterogeneity could not be found by sensitivity analysis (Supplementary Table S1). This could have been due to differences in the global cognition scoring methods and dietary questionnaires used in the studies. Specifically, Kesse-Guyot (20) used such methods as RI-48, TMT, and forward and backward digit span tasks to create a composite cognitive score and then used this score to evaluate global cognition. Wang (13) only used MoCA. Zabetian-Targhi et al. (25) used the Rey-Osterrieth Complex Figure, a delayed reproduction after 20 min and other 7 tests. Global cognitive function was calculated as the standardized mean of all tests. In theory, comprehensive neuropsychological test batteries used by Kesse-Guyot and Zabetian-Targhi are more sensitive than cognitive decline screening tests such as the MoCA used in Wang’s research to capture subtle changes in cognition in individuals with no severe deterioration of cognitive functions and with no clinical dementia. In addition, the dietary assessment method is another reason for the differences between studies since the DII scores are calculated on them. At present, the most used method is the food frequency questionnaire, and the more items, the more accurate the results are. The 24-h recall used by Kesse-Guyot is relatively less accurate.

The global cognition plot (categorical variables) in Figure 2. Also has a relatively high heterogeneity (*I*^2^ = 54%, *p* = 0.07). After excluding the paper written by Skoczek-Rubińska, the heterogeneity was reduced to 0% (*p* = 0.39) with a substantially unchanged effect size (OR = 1.53; 95% CI = 1.29–1.81). Although the small sample size and the single assessment method may be one of the reasons for the larger contribution to heterogeneity, we believe that the greatest feature of this article is the study of postmenopausal women as a specific population. Depletion of estrogen due to menopausal transition is also associated with a decline in cognitive health in women. This happens since estrogen benefits hippocampal and prefrontal cortical function, potentially enhancing verbal memory and executive function (38, 39). Therefore, we believed that the difference in the study population is the main reason for the high heterogeneity. But all in all, either include or exclude this article, the conclusion that higher DII, poorer global cognition does not change.

Our study also showed that high DII scores are negatively associated with verbal fluency, but there was no significant association between DII and executive function or episodic memory. Only three articles (19–21) studied the association between DII and these three cognitive domains. Song used categorical variables, while Frith and Kesse-Guyot used continuous variables. As to the meta-analysis of episodic memory (categorical variables) in Figure 2, we can find that the two data are from the same article, the participants are the same, and the evaluation method is two different parts of CERAD, so there is probably a bias. And when it comes to the meta-analysis of episodic memory (continuous variables) in Figure 3, the heterogeneity was relatively high (*I*^2^ = 63%, *p* = 0.10). However, because only two articles were included in the meta-analysis, we were unable to perform a sensitivity analysis to identify the source of heterogeneity. We can only speculate that the possible reasons for the high heterogeneity are different study populations (one was American while another was French) and different episodic memory assessment methods (one used CERAD while another used RI-48). Although few papers took these three cognitive domains into account, we did find one paper published in 2020 that did so (40). Its authors found that there was no association between CRP and verbal fluency. To some extent, this is inconsistent with our own conclusion. However, the paper’s participants were not elderly, and it only took CRP into account. There are many inflammatory cytokines other than CRP, such as TNFα, IL-6, and IL-10, that may be related to cognitive function. This could be why its conclusion differed from ours. Regarding executive function and episodic memory, one study conducted in the United States (41) suggested that TNFα had no association with executive function or episodic memory. Its findings were consistent with our conclusion. Even so, the evidence of the connection between DII and verbal fluency, executive function, and episodic memory is limited.

Several limitations of this work should be addressed. Firstly, most of the included studies were cross-sectional studies. Although we found that DII was associated with MCI and AD, further prospective cohort studies with different ethnic or age groups are still needed to confirm the results. For example, prospective cohort studies that include more young and healthy groups for long-term follow-up. Furthermore, a recently developed plasma biomarker for imaging, cerebrospinal fluid, or plasma biomarkers of amyloid and tau pathology can be used to assist in the early screening and diagnosis of MCI and AD, as well as the use of cognitive-related scales. Secondly, although there is convincing evidence from observational studies, including the evidence presented in the present meta-analysis, that a dietary intervention with an anti-inflammatory diet might be beneficial to counteract the risk of developing dementia or cognitive decline, there are no clinical trials to definitively support this result. Therefore, it is important for future research to address this issue. A pro-inflammatory diet might be a good candidate for an easy-to-implement, relatively cheap, and safe intervention that can be studied in association with dementia and cognitive decline using an experimental design. Thirdly, when conducting subgroup analysis, such as episodic memory (continuous variables, heterogeneities were high level), because fewer than three studies were included, we were unable to conduct sensitivity analysis to further identify the source of heterogeneity. Meanwhile, although current evidence suggests that inflammation has influences on cognitive function to some extent, the mechanism is still not clear. We look forward to further studies to address this issue in the future.

In conclusion, data from this study support an association between DII and increased risk of AD and MCI. A negative association was observed between DII and global cognition and verbal fluency. However, there was no significant association observed between DII and executive function or episodic memory. This study indicates that an anti-inflammatory diet can help prevent cognitive decline. However, current evidence on the role of diet-induced inflammation in different cognitive domains should be supplemented with further work in the future.

## Author contributions

DB: conceptualization, formal analysis, resources, writing – review and editing, and funding acquisition. DB, LW, SC, TD, and MA: methodology and investigation. TD and MA: writing – original draft preparation. JS: visualization. All authors contributed to the article and approved the submitted version.

## Funding

This research was funded by Medicine and Engineering Interdisciplinary Research Fund of Shanghai Jiao Tong University, grant number (YG2022QN011), Shanghai Municipal Health Commission, grant number (2020YJZX0139), Shanghai Municipal Science and Technology (22692191800) and National Key Research and Development Program of China (2021YFE0111800).

## Conflict of interest

The authors declare that the research was conducted in the absence of any commercial or financial relationships that could be construed as a potential conflict of interest.

## Publisher’s note

All claims expressed in this article are solely those of the authors and do not necessarily represent those of their affiliated organizations, or those of the publisher, the editors and the reviewers. Any product that may be evaluated in this article, or claim that may be made by its manufacturer, is not guaranteed or endorsed by the publisher.
