# Supplementary material for: Meta-analysis of the association between dietary inflammatory index and cognitive health
Source: Front Nutr. 2023 Apr 4;10:1104255. doi: 10.3389/fnut.2023.1104255 (PMC10111053; doi:10.3389/fnut.2023.1104255)
Supplement: Supplementary file 1 [file Data_Sheet_1.docx]

**Meta-Analysis of the Association between Dietary Inflammatory Index and Cognitive Health**

**Tianze Ding ^1,2,†^, Maimaitiyusupu Aimaiti ^1,2,†^, Shishuang Cui ^3^, Junhao Shen ^2^, Mengjie Lu ^4^, Lei Wang ^1^ and Dongsheng Bian^4,5,*^**

1 Department of Geriatrics, Medical Center on Aging of Shanghai Ruijin Hospital, Shanghai Jiao Tong University School of Medicine, Shanghai 200025, China; ilikesaber@sjtu.edu.cn (T.D.); mamatyusup@sjtu.edu.cn (M.A.)

2 Department of Nutrition, College of Health Science and Technology, Shanghai Jiao Tong University School of Medicine, Shanghai 200025, China; s123456@sjtu.edu.cn (J.S.)

3 Department of Geriatrics, Ruijin Hospital, School of Medicine, Shanghai Jiao Tong University, Shanghai 200025, China; csx01b03@rjh.com.cn (S.C.)

4 School of Public Health, Shanghai Jiao Tong University School of Medicine, Shanghai 200025, China; lu_mengjie@sjtu.edu.cn (M.L.); bds04159@rjh.com.cn (D.B.)

5 Department of Clinical Nutrition, Ruijin Hospital, Shanghai Jiao Tong University School of Medicine, Shanghai 200025, China;

* Correspondence: bds04159@rjh.com.cn (D.B.); [wl10779@rjh.com.cn](mailto:wl10779@rjh.com.cn) (L.W.)

† These authors contributed equally to this work.

Supplemental table1

Sensitivity analysis of Global Cognition. Note: continuous variables.

| Excluded paper | OR (95% CI) (fixed) | OR (95% CI) (random) | I^2^ | P |
| --- | --- | --- | --- | --- |
| Wang 2022 | 0.98 (0.80, 1.20) | 0.46 (0.08, 2.69) | 91% | < 0.01 |
| Kesse-Guyot 2016 | 0.89 (0.76, 1.05) | 0.86 (0.58, 1.29) | 83% | = 0.02 |
| Zabetian-Targhi 2021 | 0.64 (0.50, 0.83) | 0.38 (0.10, 1.48) | 85% | = 0.01 |

Supplemental figure1

Sensitivity analysis of Global Cognition (exclude Skoczek-Rubińska 2021). Note: categorical variables.
